# Tattoo-Associated Skin Reaction: The Importance of an Early Diagnosis and Proper Treatment

**DOI:** 10.1155/2014/354608

**Published:** 2014-07-23

**Authors:** Andrea Bassi, Piero Campolmi, Giovanni Cannarozzo, Rossana Conti, Nicola Bruscino, Massimo Gola, Stefano Ermini, Daniela Massi, Silvia Moretti

**Affiliations:** ^1^Department of Surgery and Translational Medicine, Section of Dermatology, University of Florence Medical School, Hospital P. Palagi, 50125 Florence, Italy; ^2^Department of Immunology and Allergology, University of Florence Medical School, 50125 Florence, Italy; ^3^Division of Pathological Anatomy, University of Florence Medical School, 50125 Florence, Italy

## Abstract

Tattoo is going to be a very common practice especially among young people and we are witnessing a gradual increase of numerous potential complications to tattoo placement which are often seen by physicians, but generally unknown to the public. The most common skin reactions to tattoo include a transient acute inflammatory reaction due to trauma of the skin with needles and medical complications such as superficial and deep local infections, systemic infections, allergic contact dermatitis, photodermatitis, granulomatous and lichenoid reactions, and skin diseases localized on tattooed area (eczema, psoriasis, lichen, and morphea). Next to these inflammatory skin reactions we have to consider also the possibility of the development of cutaneous conditions such as pseudolymphomatous reactions and pseudoepitheliomatous hyperplasia. The aim of this study is to underline the importance of an early diagnosis by performing a histological examination especially when we are in front of suspected papulonodular lesions arising from a tattoo, followed by a proper treatment, since cutaneous neoplastic evolution is known to be a rare but possible complication.

## 1. Introduction

The number of tattooed people has become more prevalent in the last few years for the continuous development of new trends especially among young people. Despite the increasing number of tattooed individuals, there are currently few requirements, little legislation, and few criteria for the safety of tattoos. In Italy, composition of tattoo inks is not regulated by law and their labeling is not compulsory [1]. Frequently, there is no information on packaging, such as expiration date, conditions of use, warnings, or the guarantee of sterility of the contents [2]. Consequently, we are witnessing a gradual increase of numerous potential complications to tattoo placement which are often seen by physicians, but generally unknown to the public [3].

The most common skin reactions to tattoo reported in the literature [4–7] include a transient acute inflammatory reaction due to trauma of the skin with needles and medical complications such as superficial and deep local infections, systemic infections, allergic contact dermatitis, photodermatitis, granulomatous and lichenoid reactions, and skin diseases localized on tattooed area (eczema, psoriasis, lichen, and morphea) (Table 1). These reactions may have different onset of symptoms immediately when the tattoo has been inked into it, days, months, or years later [8–10]. Next to these inflammatory skin reactions we have to consider also the possibility of the development of cutaneous conditions such as pseudolymphomatous reactions [11] and pseudoepitheliomatous hyperplasia [12]. The evolution in neoplastic lymphoma [13, 14], squamous cell carcinoma, and Keratoacanthoma [15] is a rare outcome, since this neoplastic condition usually appears when they are fully evolved and not with “premalignant” condition. Despite that, it is mandatory to perform histological examination when we are in front of suspected papulonodular lesions arising from a tattoo.

The aim of this study is to underline the importance of an early diagnosis and proper treatment of tattoo reactions, since cutaneous neoplastic evolution is known to be a rare but possible complication, even if this process was not found in our experience.

## 2. Methods

During the period from May 2012 to April 2013, a total of 16 patients (6 women, 10 men) were treated at the Department of Dermatology in Florence for the development of cutaneous reactions secondary to tattoos. All these patients underwent allergological examination by means of patch tests according to the standard SIDAPA series (Italian Society of Allergological, Occupational and Environmental Dermatology) (Table 2(a)) and the International Contact Dermatitis Research Group guidelines, using the IQ chamber method (Chemotechnique Diagnostics, Vellinge, Sweden). Standard allergological examination was supplemented with various metal haptens associated with different tattoo pigments (Table 2(b)). Five patients were tested directly with the pigment used for the tattoo, while it was impossible for the others. A punch biopsy of the skin was performed in all patients for histopathological examination. Cutaneous reactions were documented with photographs taken by a Canon digital camera.

## 3. Results

**Figure 1:**
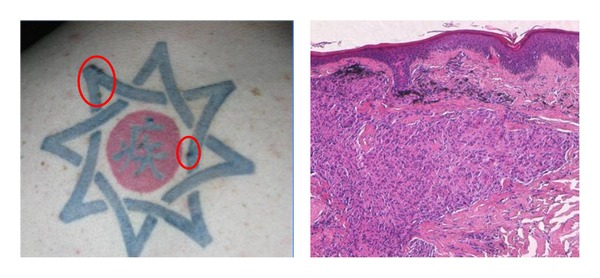
Foreign body granulomatous reaction on a black tattoo and relative histology with granulomatous depositions of exogenous pigment in the background of granulomatous-productive inflammation of the dermal stroma.

**Figure 2:**
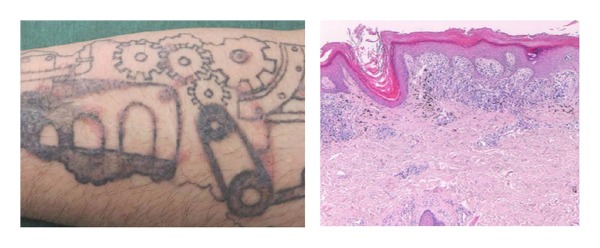
Lichenoid reaction on a black tattoo and relative histology with a widespread vacuolar basal epidermic degeneration with a deep dermal lymphohistiocytic infiltrate into a lichenoid pattern, associated with deposition of exogenous pigment.

**Figure 3:**
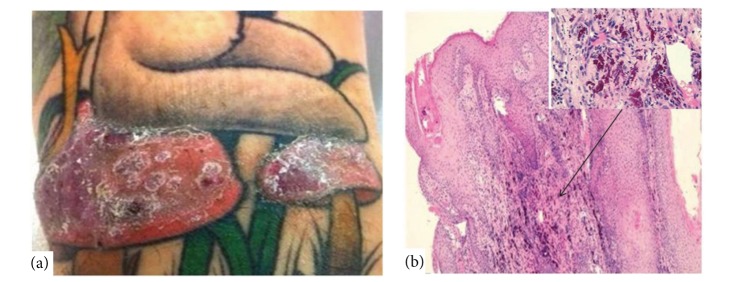
(a) Pseudoepitheliomatous hyperplasia on a red tattoo and relative histology with epidermal pseudoepitheliomatous hyperplasia and follicular hyperkeratosis (H&E, original magnification ×5); (b) the part indicated by the arrow shows an inflammatory infiltrate in the dermis, composed of lymphocytes and plasma cells and dermal exogenous pigment deposition (H&E, original magnification ×40).

**Figure 4:**
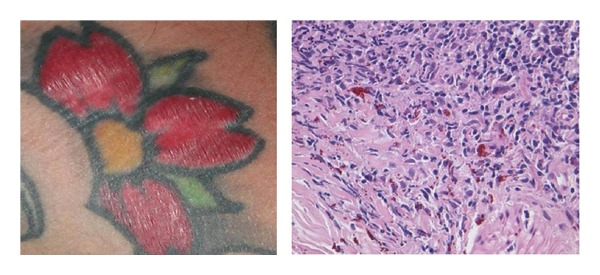
Pseudolymphomatous reaction on a red tattoo and relative histology with the presence of red colour exogenous pigment in the background of a reactive lymphoid hyperplasia in the superficial and medium dermis.

**Figure 5:**
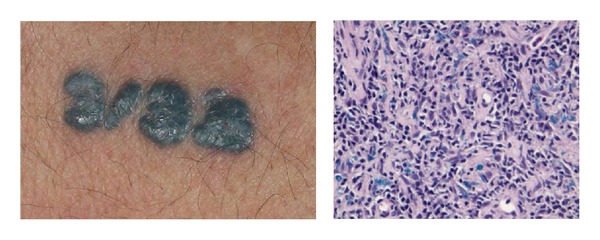
Pseudolymphomatous reaction on a black tattoo and relative histology with the presence of black colour exogenous pigment in the background of a reactive lymphoid hyperplasia in the superficial and medium dermis.

In the above-mentioned period for the 16 patients, the reactions to tattoo were 5 foreign body granulomatous reactions (Figure 1), 4 lichenoid reactions (Figure 2), 2 psoriasis, 1 pseudoepitheliomatous hyperplasia (Figure 3), and 4 pseudolymphomatous reactions of which 3 were on red tattoos and 1 was on a black tattoo (Figures 4 and 5). All the 16 patients were negative for allergological examination with the standard SIDAPA series and the tattoo additional one. Patch testing with the real pigment used for the tattoo in 5 patients was also negative. Histological examination for the foreign body granulomatous reactions revealed granulomatous depositions of exogenous pigment in the background of granulomatous-productive inflammation of the dermal stroma. The lichenoid reactions showed a widespread vacuolar basal epidermic degeneration with a deep dermal lymphohistiocytic infiltrate into a lichenoid pattern, associated with deposition of exogenous pigment. The pseudolymphomatous reactions demonstrated the presence of colour (red or black) exogenous pigment in the background of a reactive lymphoid hyperplasia in the superficial and medium dermis. The specimens were analysed by PCR-based IgH chain (FR2A-JH and FR3A-JH segments), T-Cell Receptor gamma and T-Cell Receptor beta clonality detection, and heteroduplex analysis of PCR products revealing polyclonal rearrangement of these regions. Polarizing microscopy showed no signs of birefringence. The pseudoepitheliomatous hyperplasia on the red tattoo showed an epidermal pseudoepitheliomatous hyperplasia and follicular hyperkeratosis with an exogenous red pigment deposition which was demonstrated within the dermis, associated with a mixed inflammatory infiltrate, composed of lymphocytes and plasma cells.

**Figure 6:**
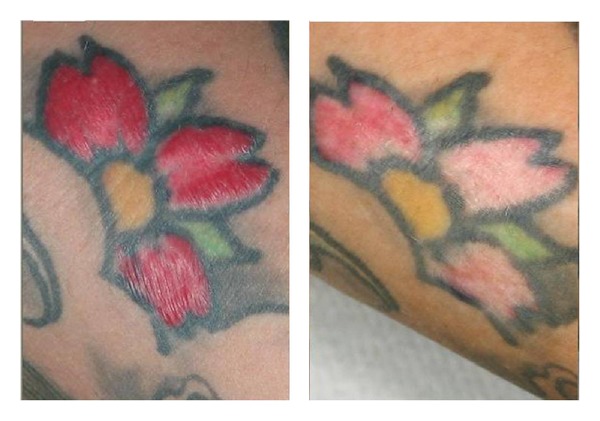
Resolution of the lesions of pseudolymphoma on the red portion of the tattoo after 4 sessions of Q-Switched Nd:YAG laser.

Patients with inflammatory skin reaction (granulomatous, lichenoid, and psoriasis) were treated with topical steroid with rapid improvement of skin lesions. Patients with neoplastic reaction (pseudoepitheliomatous hyperplasia and pseudolymphoma) initially underwent systemic steroid treatment (methylprednisolone acetate 40 mg PO daily) resulting into complete clearance of the lesions, but they reappeared immediately after ten days. Subsequent treatment with Q-Switched Nd:YAG laser (DEKA M.E.L.A, srl, Calenzano, Italy) resulted into almost complete lesion disappearance (Figure 6). Q-Switched Nd:YAG laser was used in two different wavelengths at 1064 nm with a spot size of 3 mm and a fluence of 11–12.5 J/cm^2^ and 532 nm with a spot size of 3 mm and a fluence of 3 J/cm^2^.

## 4. Discussion

Tattooing has recently become increasingly popular and not only among young people, giving rise to parallel increase of adverse reactions. With the increasing incidence of tattooing fashion trend in society, physicians should be able to recognize tattoo complications and also appropriately counsel their patients on risks of tattoo placement [16]. Numerous potential complications secondary to tattoo placement have been reported in literature. Tattoo reactions can be divided into three main categories: inflammatory, infectious, and neoplastic. Inflammatory manifestations include focal oedema, pruritus, papules, or nodules at the tattoo site. Histologically they can be classified as lichenoid, eczematoid, foreign body granulomatous, and sarcoidal [17]. Less commonly, psoriasiform, morpheaform, and vasculitic reaction have also been reported [18, 19]. The infectious reaction can be generally distinguished in bacterial, viral or mycotic and they can appear as superficial or with deep skin involvement. Rare but possible reactions are represented by pseudoepitheliomatous hyperplasia and pseudolymphoma. They are not considered as real premalignant conditions. The neoplastic complication described within tattoo pigments includes keratoacanthoma, squamous cell and basal cell carcinoma, leiomyosarcoma, and melanoma. When we observe tattoo malignancies, usually squamous cell carcinoma, they are fully evolved and they do not present as “premalignant" condition. According to the literature, the most frequent tattoo reactions concern allergic contact dermatitis due to delayed hypersensitivity reaction to different pigments contained in the tattoos [20, 21]. The main pigment causing allergic reaction is the red one, due to the presence of mercury and its sulphides [22]. However, nowadays most reactions are not due to the traditional presence of mercury sulphides, but due to new organic pigments (e.g., Pigment Red 181 and Pigment Red 170) [1]. Different from red tattoo, the blue, green, and black tattoos are a less frequent cause of allergic contact dermatitis [23–26]. Actually allergological reactions to temporary henna tattoos due to the* para*-phenylenediamine are very common [27]. The first conclusion of our study showed that the results of these 16 patients for standard series and additional and specific haptens were negative. Therefore we noticed that allergic reactions due to sensitization to haptens contained in tattoos are not very frequent. A possible explanation is that needles used by tattoo artists, passing through the skin and inoculating haptens directly in the dermis, could bypass the normal mechanism of hapten processing. In fact, the first phase of the hapten processing usually occurs in the epidermis, where antigen presenting cells are present in great number, whereas the inoculation of the hapten in the dermis without any adjuvant could escape the first phase of the innate immunity. For example, we can suggest that whereas a typical hypersensitivity reaction, such as the one directed towards p-phenylenediamine, is possibly due to the activation of memory T cells previously activated by occasional contact (i.e., hair dye), it is unlikely that we could have been in contact with substances present in tattoo inks.

Even the most recent subcutaneous vaccinations (such as the flu vaccine) need the presence of adjuvants. Moreover, the nature of the antigen is different, viral particles being more immunogenic than haptens.

Another hypothesis is that tattoo composition is very heterogeneous and in continuous evolution (Table 3), and we have tested solely the SIDAPA standard series and only few metal haptens associated with different tattoo pigments.

The second conclusion is related to the possible development of neoplastic skin manifestation arising from a tattoo. Four of the 16 patients presented a histological examination compatible with a pseudolymphomatous reaction and one patient presented a pseudoepitheliomatous hyperplasia reaction. The pathogenesis of malignancies in tattoos is far from being obvious since it is not clarified if the development of neoplasms in tattoos is coincidental or is somehow due to the tattoo. The trauma induced by the procedure (puncturing the skin) has always been pointed out in the literature as one of the main causes: tattoo pigments do not remain inert in the dermis because a skin inflammatory reaction occurs during the whole life in an attempt to degrade the foreign material, and it is unknown exactly what type of reactions occurs [28]. This inflammatory reaction starts as being local but can become generalized. Trauma is probably not the only factor, since tattoo inks have been shown to contain numerous potentially hazardous and carcinogenic compounds that hypothetically could be tumorigenic [29]. However, the potential carcinogenicity or toxicity related to the pigments/dyes or their byproducts still remains unclear [30]. Unfortunately, only in vitro data are available showing that some azo dyes, under certain circumstances (laser therapy, UV exposure), may lead to the increase of some carcinogenic substances such as 3,3-dichlorobenzidine [30, 31].

An early diagnosis through skin biopsy, especially with papulonodular growth within the tattoo pigment, is mandatory since neoplastic conditions are not immediately recognized with clinical examination only. For that reason, the transformation from a pseudolymphoma into a lymphoma and the transformation from a pseudoepitheliomatous hyperplasia into a squamous cell carcinoma or keratoacantoma are very rare, but not impossible [13–15].

Once a diagnosis has been established, it is important to remove the lesion. For refractory skin eruptions unresponsive to medical therapy, surgical or laser treatment may be considered. In our patients we have used a Q-Switched Nd:YAG laser capable of emitting two different wavelengths at 1064 nm (useful for dark blue and black pigment) and 532 nm (useful for removal of red, orange, and purple tattoo pigments). The Q-Switched Nd:YAG system releases high energy in extremely short times (max 6 ns), producing a “photoacoustic” effect that breaks down the derma cells containing the tattoo pigment. Thanks to the rupturing of the membrane of these cells, the pigment is released and eliminated by the lymphatic system. These short laser emissions allow for confining the thermal effect exclusively to the target—in this case the tattoo pigment—therefore safeguarding the surrounding tissues [32, 33]. Moreover, laser systems allow the best aesthetic results without leaving scars if compared with surgery that sometimes may be more radical but capable of injuring the underlying tissue.

## 5. Conclusion

The medical literature contains numerous case reports on dermatological reaction after tattoos procedure. Our experience on a small number of patients underlines that the allergic contact reaction is not the most frequent complication after the tattoo procedure. The potential correlation between tattooing and skin cancer is rare but not impossible, though its pathogenesis is still unclear. Our study is subject to several limitations due to the number of patients involved. Further studies should be done to investigate in depth the possible evolution to malignancy. A follow-up of a large cohort of tattooed people would help to assess whether or not tattooing is an independent risk factor for skin malignances. This paper should advise not only dermatologist but also physicians about the possible risk of malignancies in tattoo reactions. In suspected cases, especially in front of papulonodular lesions arising from a tattoo, it is important to perform an early diagnosis through histopathology followed by removal of the lesion.

## Figures and Tables

**Table 1:** Dermatologic disorders and complications after tattooing.

Complications after tattooing	Clinical features	Onset of symptoms
Allergic disorders	Allergic dermatitis	Days to weeks
	Photoallergic reaction	After sun exposure

Skin infections	Erysipelas	First few days
	Gangrene	=
	Sepsis	=
	Impetigo	=
	Ecthyma	=
	Cellulitis	=
	Tetanus	Weeks to years
	Lepra	=
	Syphilis	=

Viral infections	Molluscum contagiosum	Weeks to months
	Verruca vulgaris	=
	Hepatitis B, C	=
	AIDS	=

Mycoses	Tinea cutis glabrae	After weeks
	Zygomycoses	After months

Tumors	Lymphoma	Years
	Carcinoma basocellular	=
	Carcinoma spinocellular	=
	keratoacanthoma	=
	Melanoma	=

Skin disease localized in tattooed area	Psoriasis	Weeks to years
	Lichen planus	=
	Morphea	=
	Pseudolymphoma	=
	Pseudoepitheliomatous hyperplasia	=

**(a):** 

Thiuram mix	1% pet.
Potassium dichromate	0.5% pet.
Balsam Peru	25% pet.
Phenylisopropyl-p-phenylenediamine	0.1% pet.
Kathon CG	0.01% aqua
p-Phenylenediamine	1% pet.
Lanolin alcohol	30% pet.
Colophony	20% pet.
Neomycin sulfate	20% pet.
Cobalt chloride	1% pet.
Epoxy resin	1% pet.
Formaldehyde	1% aqua
Mercaptobenzothiazole	2% pet.
p-ter-Butylphenol-formaldehyde resin	1% pet.
Nickel sulphate	5% pet.
Disperse yellow 3	1% pet.
Fragrance mix + sorbitan sesquioleate	8% pet.
Paraben mix	16% pet.
Disperse blue 124	1% pet.
Benzocaine	5% pet.
Dibromodicyanobutane	0.3% pet.
Corticosteroid mix	2.01% pet.
Lyral	5% pet.
Mercapto mix	2% pet.
Desoximetasone	1% pet.

**(b):** 

Cadmium chloride	1% pet.	Yellow
Chromium oxide	2% aqua	Green
Mercury metal	0.5% pet.	Red
Copper sulfate	1% aqua	Blue
Ferric oxide	2% pet.	Black
Aluminium chloride	2% pet.	Purple
Zinc metal	1% pet.	White

**Table 3:** Other possible ingredients and haptens contained in the tattoo ink and pigment colour.

Tattoo ink/pigment color	Ingredient
Black	Iron oxide
	Carbon
	Logwood

Brown	Ochre (ferric oxide)

Red	Cinnabar/mercuric sulfide
	Cadmium red
	Iron oxide/common rust
	Napthol-AS pigment

Yellow	Cadmium yellow
	Ochre
	Curcuma yellow
	Chrome yellow (PbCrO_4_, often mixed with PbS)

Green	Chromic oxide (Casalis Green or Anadomis Green)
	Lead chromate
	Phthalocyanine dyes
	Ferrocyanides and ferricyanides

Blue	Azure blue
	Cobalt blue
	Cobalt phthalocyanine
	Cobalt aluminate

Violet (purple)	Manganese ammonium pyrophosphate
	Various aluminum salts
	Dioxazine/carbazole

White	Lead carbonate
	Titanium dioxide
	Barium sulfate
	Zinc oxide

Henna	Henna dye and paraphenylenediamine (PPD)
